# Tetramycin ameliorates tebuconazole·azoxystrobin to control leaf spot and viral diseases of Taizishen

**DOI:** 10.3389/fpls.2025.1543462

**Published:** 2025-02-04

**Authors:** Bing Tian, Chenglin Tang, Jiaqi Liu, Boya Jin, Cheng Zhang

**Affiliations:** ^1^ Key Laboratory of Environmental Pollution Monitoring and Disease of Ministry of Education, School of Public Health, Guizhou Medical University, Guiyang, Guizhou, China; ^2^ Guizhou Agricultural Ecology and Resource Protection Station, Agriculture and Rural Affairs Department of Guizhou Province, Guiyang, Guizhou, China; ^3^ Guizhou Crop Technology Extension Station, Agriculture and Rural Affairs Department of Guizhou Province, Guiyang, Guizhou, China

**Keywords:** agricultural antibiotic, electrophysiological information, leaf spot and disease, viral disease, growth and quality, *Pseudostellaria heterophylla*

## Abstract

Leaf spot and viral diseases are the most frequently occurring leaf problems in Taizishen production. In this study, we examined the controlling role played by the co-application of tetramycin and low dose tebuconazole·azoxystrobin against leaf spot and viral diseases in Taizishen, investigating its resistance, electrophysiological information, growth and quality. Among them, electrophysiological information indicators include electrical signals [intrinsic capacitance (IC), resistance (IR), impedance (IZ), capacitive reactance (IXc), and inductive reactance (IXL)], intracellular water metabolism, nutrient transport, and plant metabolic activity. The results indicate that 0.3% tetramycin 1000-time + 75% tebuconazole·azoxystrobin 2000-time diluent controlled leaf spot and viral diseases the best, with protection effects of 90.03%~90.46% and 71.67%~73.08% at 15~30 days after the last fungicide application, respectively. These values are obviously higher than those treated with high doses of tetramycin or tebuconazole·azoxystrobin alone. Concurrently, their combined application could notably enhance total soluble flavonoids, total soluble phenols, protective enzyme activity, IC, intracellular water metabolism, nutrient transport, and metabolic activity, while reducing its MDA, IR, IZ, IXc, and IXL. Moreover, their co-application also could obviously ameliorate photosynthesis, biomass, agronomic trait, and root growth and quality, as well as actually reduce tebuconazole·azoxystrobin input. Additionally, the control effects of leaf spot and viral diseases in Taizishen treated by their combined application exhibited significant correlations with its disease resistance, electrophysiology, photosynthesis, growth, and quality parameters. This study highlights the combined application of low-dosage tebuconazole·azoxystrobin and tetramycin as a practicable measure for controlling leaf spot and viral diseases in Taizishen, promoting its resistance, growth, and quality, as well as reducing chemical pesticide application.

## 1 Introduction

*Pseudostellaria heterophylla*, also known by its Chinese name, Taizishen, is an edible, medicinal, and ornamental plant. It has a long history in traditional Chinese food and medicine for its multiple pharmacological functions, such as enhancing immunity, improving blood quality, moistening the lungs, inhibiting tumor cells, protecting myocardial function, invigorating the spleen–stomach, and preventing and curing COVID-19 (Choi et al., 2017; Shi et al., 2020; Wang et al., 2013; Zhang et al., 2016, 2021). Meanwhile, its roots, which are rich in polysaccharides, saponins, amino acids, flavonoids, and minerals, have high nutritious, medicinal, and economical value (Liao et al., 2018; Ma et al., 2022; Xu et al., 2023; Zhang et al., 2021). It is predominantly produced in China, the Korean Peninsula, the far east of Russia, and Japan (Liao et al., 2018). Recently, it has been popularly cultivated in the provinces of Guizhou, Fujian, Zhejiang, Sichuan, and Anhui in China, especially in Guizhou, where the cultivating area of over 20, 000 hm^2^ ranks first in the country (Wu et al., 2016). Nevertheless, leaf spot disease caused by various pathogens, such as *Alteraria tenuissima*, *Arcopilus versabilis*, *Phyllosticta commonsii*, etc., and viral disease caused by *Turnip mosaic virus*, *Broad bean wilt virus*, *Tobacco mosaic virus*, *Cucumber mosaic virus*, etc., are most frequently occurring leaf diseases during Taizishen growth (He et al., 2021; Long et al., 2013; Li et al., 2018; Liang et al., 2022; Yang et al., 2023a, 2023). Leaf spot and viral diseases often occur from March to May, seriously restricting plant growth, root growth and quality, and the industry’s development, as well as causing consistent economic losses of over 50% (Yang et al., 2023a). Thus, there is an urgent need to exploit a variety of potential practicable and environmentally friendly control strategies for leaf spot and viral diseases in Taizishen.

Generally, chemical fungicides are very effective and frequently adopted approaches for controlling plant diseases due to their low toxicity and high efficiency (Wang et al., 2022). Some chemical fungicides have been screened by scholars and applied for controlling Taizishen diseases, such as difenoconazole, pyraclostrobin, azoxystrobin, flusilazole, and tebuconazole (He et al., 2021; Long et al., 2013; Li et al., 2013, 2018; Shao et al., 2016; Yang et al., 2023a). For example, Shao et al. (2016) showed that 50% azoxystrobin water dispersible granule (WDG) exerted a controlling effect of 74.50%~76.61% on leaf spot disease in Taizishen. He et al. (2021) reported that a 35% triflumizole·tebuconazole suspension concentrate exhibited good antifungal activity on *A. tenuissima* with an EC_50_ value 36.10 μg ml^−1^. Tebuconazole, a triazole fungicide that is extensively applied for controlling numerous plant fungi diseases, can inhibit the sterol biosynthesis of pathogens (Fustinoni et al., 2014; Paul et al., 2008; Liu et al., 2015). And azoxystrobin is a mitochondrial respiration inhibitor fungicide which has broad-spectrum systemic activity against many fungal pathogens (Adetutu et al., 2010; Rodrigues et al., 2013; Wang et al., 2016). However, it is widely known that pesticide residues are common after chemical fungicide application; these, in turn, pose potential risks to the environment, organisms, and humans (Wang et al., 2022). Accordingly, there has been a decline in the use of chemical pesticides that has been welcomed by the public. Notably, our two most recent works suggested that chitosan could enhance low-dosage difenoconazole in controlling Taizishen leaf spot disease and that oligochitosan as a potential synergist to promote the effects of pyraclostrobin on Taizishen leaf spot; these two measures have also helped in effectively reducing chemical pesticide application (Zhang et al., 2023, 2024). In seeking more options for preventing and controlling Taizishen leaf spot and viral diseases, a long-term concern consists in finding natural products that can enhance pesticides, reduce their application, and alleviate the pathogen resistance caused by long-term single-pesticide application.

Tetramycin, a novel natural, medical, and agricultural antibiotic metabolized by *Streptomyces hygrospinosus* var. Beijingensis, has two active compositions: tetramycin A and tetramycin B (Bo et al., 2012; Ren et al., 2014). Many studies have demonstrated that tetramycin could be widely applied for preventing and controlling various plant diseases infected by fungal and bacterial, including *Alternaria tenuissima*, *Botrytis cinerea*, *Pyricularia oryzae*, *Alternaria alternata*, *Colletotrichum scovillei*, *Phytophthora capsici*, *Pseudomonas syringae* pv. *Pseudomonas fulva*, and *Agrobacterium tumefaciens* etc (Chen et al., 2017; Gao et al., 2018; Song et al., 2016; Li et al., 2023; Ma et al., 2017, 2018; Wang et al., 2021a; Zhao et al., 2010). In China, tetramycin has also been found to control crop, fruit, and vegetable diseases, and is increasingly becoming a preferred adjuvant or alternative to chemical pesticides or conventional antibiotics (Li et al., 2014; Wang et al., 2021a). Recently, Wang et al. (2021a) found that tetramycin exhibited good antimicrobial activity against kiwifruit pathogens including *Pseudomonas syringae* pv. *Botryosphaeria dothidea*, *Pseudomonas fulva*, *Alternaria tenuissima*, etc. Subsequently, they found that chitosan could augment tetramycin’s effect on soft rot disease in kiwifruit, including its effects on growth and quality (Wang et al., 2021b). Moreover, our recent works indicated that the joint application of tetramycin and chitosan or matrine could effectively control leaf spot or soft rot diseases by enhancing kiwifruit resistance, photosynthesis, and quality (Zhang et al., 2022a, 2022b). In this way, further research is needed to determine whether tetramycin can improve the controlling effect of tebuconazole·azoxystrobin against leaf spot and viral diseases in Taizishen, as well as whether their joint application could become another effective means of controlling disease and reducing the use of pesticides.

In the work, we first evaluated the field control efficacy of tebuconazole·azoxystrobin and tetramycin, as well as their formulas, in leaf spot and viral diseases in Taizishen. We simultaneously investigated the plant’s disease resistance, electrophysiological information, leaf photosynthesis, and growth. Subsequently, its root growth and quality were also determined. This work provides another green, efficient, and environmentally friendly measure for controlling leaf spot and viral diseases in Taizishen.

## 2 Materials and methods

### 2.1 Tebuconazole·azoxystrobin and tetramycin

75% Tebuconazole·azoxystrobin (TA) water dispersible granule (WDG) was obtained from Sipcam Chemical Trading (Shanghai) Co. Ltd. (Shanghai, China), it contains 50% tebuconazole and 25% azoxystrobin. 0.3% tetramycin (TE) aqueous solutions (AS) was produced by Liaoning Microke Biological Engineering Co. Ltd. (Liaoning, China).

### 2.2 Field Taizishen orchard

Field experimental orchard of Taizishen with ‘Guisheng 1’ of the planting cultivar was located in Shibing County, China (27°16′ N, 107°97′ E). In the last two years, the leaf spot and virus diseases of Taizishen in this orchard were serious, and the natural incidence rates were about 40%~50% and 10%~20%, respectively. According to the reports of our cooperative research group, *Alternaria tenuissima* was the main pathogen of Taizishen leaf spot disease in this region, and *Turnip mosaic virus* and *Broad bean wilt virus* were the main pathogens of Taizishen virus disease (He et al., 2021; Liang et al., 2022). In the year of field experiment, the leaf spot and virus diseases were natural occurrence. Riding planting of Taizishen seed roots in this orchard, the plot area is 6.0 m^2^ (2.0 m of length, 3.0 m of width, 0.2 m in between), and the seed cultivating density was 30 kg per 667 m^2^. Moreover, its soil fertility are shown in **Table 1**.

**Table 1:** The soil fertility of Taizishen orchard.

Fertility	Content (g kg^−1^)	Fertility	Content (mg kg^−1^)
Organic matter	37.13	Available phosphorus	57.02
Total nitrogen	1.57	Available potassium	131.35
Total phosphorus	1.72	Available iron	8.33
Total potassium	1.23	Exchangeable magnesium	291.65
pH	4.96	Available zinc	2.01
Exchangeable calcium	21.04 cmol kg^−1^	Available manganese	18.33
Alkali hydrolyzed nitrogen	115.63 mg kg^−1^	Available boron	0.22

### 2.3 Field control experiment

A completely randomized method was applied for delineating plots and leaf spraying method was applied for spraying fungicide. Six treatments were designed: (1) 75% tebuconazole·azoxystrobin WDG 2000-time + 0.3% tetramycin AS 1000-time diluent (TA 2000+TE 1000), (2) 75% tebuconazole·azoxystrobin WDG 1500-time diluent (TA 1500), (3) 75% tebuconazole·azoxystrobin WDG 2000-time diluent (TA 2000), (4) 0.3% tetramycin AS 500-time diluent (TE 500), (5) 0.3% tetramycin AS 1000-time diluent (TE 1000), and (6) clear water with non-fungicide (Control). Meanwhile, each treatment contained three replicate plots, and fungicide diluent was sprayed on Taizishen aboveground parts by an electrostatic atomizer. The spraying dates were March 28, April 8, and April 18, respectively, and the application diluent amount each time was 60 L per 667 m^2^.

### 2.4 Investigation methods

#### 2.4.1 Control effect determination

Leaf spot and virus diseases of Taizishen were natural infections in the test area, and they were identified via symptom recognition method referred to He et al. (2021) and Liang et al. (2022). Leaf spot disease: In the early stage of leaf spot infection, the affected area of the leaves showed yellow spots with white center, light yellow edges, and water stained; As the infection time prolongs, the disease spot gradually expands to form a yellow brown withered spot; In the later stage of infection, the spots showed a circular pattern with black smalldots (conidia), and the whole leaves died under severe cases. Viral disease: When the disease was mild, the leaf veins became lighter and yellowish, often formed flower leaves with alternating shades of light and dark; When the disease was severe, the leaves were wrinkled and showed spots, the leaf edges were often curled, and large necrotic spots appeared on the infected leaves.

The disease index of leaf spot disease and incidence rate of viral disease were investigated on May 3 (15 days after the last spraying fungicide) and May 18 (30 days after the last spraying fungicide). One hundred plants were chosen from the east, south, west, north, central parts in every plot, and their diseased leaf and plant number of leaf spot and viral diseases were counted. The classification of diseased leaf rank for leaf spot disease:

0 rank: no spot;
1 rank: the spot extent was lower than 5% of total leaf extent;
3 rank: the spot extent was 6% to 10% of total leaf extent;
5 rank: the spot extent was 11% to 20% of total leaf extent;
7 rank: the spot extent was 21% to 50% of total leaf extent;
9 rank: the spot extent was more than 51% of total leaf extent.

Then, the disease index and control effect for leaf spot disease were determined as:


(1) $$\begin{array}{c} \text{Disease index}=100\times \sum \;(\text{Leaf number of each rank}\times \\ \text{Rank value})/(\text{Highest rank value}\\ \times \text{Leaf total number}) \end{array}$$

(2) $$\begin{array}{c} \text{Control effect }\left(\%\right)\text{of leaf spot disease}\\ =100\times (1\text{-Disease index of fungicide treatment}\\ /\text{Disease index of non-fungicide treatment}) \end{array}$$

Simultaneously, the incidence rate and control effect for viral disease was calculated as:


(3) $$\begin{array}{c} \text{Incidence rate of viral disease }\left(\%\right)\\ =100\times (\text{Number of infected plants}\\ /\text{total number of checked plants}) \end{array}$$

(4) $$\begin{array}{c} \text{Control effect }\left(\%\right)\text{ of viral disease}\\ =100\times (1\text{-Incidence rate of fungicide treatment}/\\ \text{Incidence rate of non-fungicide treatment}) \end{array}$$

#### 2.4.2 Leaf resistance determination

Five Taizishen leaves were randomly sampled from the east, south, west, north, central parts in each plots on May 18 and brought their back to the laboratory for checking disease resistance levels according to Zhang et al. (2022c). Accordingly, thiobarbituric acid, rutin standard curve, and gallic acid standard curve methods were used for measuring malonaldehyde (MDA), total soluble flavonoids, and total soluble phenols conments, respectively. For MDA determination, 1.00 g sample was added into 5 mL 10% trichloroacetic acid ice bath to grind into homogenate, centrifuged; 2 mL supernatant was added into 2 mL 0.6% thiobarbituric acid solution, boiled at water bath (15 min), cooled and centrifugated, measured at OD _450 nm_ and OD _532 nm_, and 10% trichloroacetic acid as control. For total soluble flavonoids and total soluble phenols determination, 2.00 g sample was added into 20 mL 1% HCl-methyl alcohol (v/v) for extracting 1 h without light and centrifuged, then the supernatant was checked at OD _325 nm_ and OD _280 nm_, rutin and gallic acid as control, respectively. Moreover, their superoxide dismutase (SOD), phenylalaninammo nialyase (PAL), peroxidase (POD), and polyphenoloxidase (PPO) activities were analyzed by nitrogen blue tetrazole, trans-cinnamic acid, guaiacol, and catechol methods, respectively. Briefly, the appropriate amount sample was extracted by corresponding reagents and centrifuged, the supernatant was measured at OD _560 nm_, OD _290 nm_, OD _470 nm_, and OD _398 nm_ for checking SOD, PAL, POD, and PPO activities, respectively.

#### 2.4.3 Electrophysiological information determination

Five Taizishen plants on the east, south, west, north, central parts in every plot were randomly selected for monitoring their electrophysiological information on May 18 according to the methods of Zhang et al. (2020, 2021a, 2021b) and Tian et al. (2024). In our previous studies, the theoretically intrinsic relationships between the clamping force and leaf R, Z, Xc or XL were revealed as R, Z, Xc or XL =y+k e^(-bF) based on the Nernst equation (Zhang et al., 2020, 2021a, 2021b). When the clamping force is 0 (F=0), then the intrinsic R, Z, Xc and XL of plant leaves could be monitored as IR, IZ, IXc or IXL =y+k, and intrinsic capacitance (IC) was calculated according to formula:


(5) $$\text{IC}=\frac{1}{2\text{πfIXc}}$$

where π=3.1416, f= frequency.

Moreover, the intracellular water-holding capacity (IWHC), intracellular water-holding time (IWHT), water or nutrient transfer rate (WTR or NTR), nutrient flux per unit area (UNF), nutrient transport capacity (NTC), the active transport flow of nutrient (NAF), nutrient active transport capacity (NAC), metabolic flow (MF), metabolic rate (MR), and metabolic activity (MA) were calculated as (Tian et al., 2024):


(6) $$\text{IWHC}=\sqrt{{\text{(IC)}}^{3}}$$

(7) IWHT=IC×IZ

(8) $$\text{WTR or NTR}=\frac{\text{IWHC}}{\text{IWHT}}$$

(9) $$\text{UNF}=\frac{\text{IR}}{\text{IXc}}+\frac{\text{IR}}{\text{IXL}}$$

(10) NTC=UNF×NTR

(11) $$\text{UAF}=\frac{\text{IXc}}{\text{IXL}}$$

(12) NAC=UAF×NTR

(13) $$\text{MF}=\frac{1}{\text{IR}\times \text{IZ}\times \text{IXc}\times \text{IXL}}$$

(14) MR=NTR×NAC

(15) $$\text{MA}=\sqrt[{6}]{\text{MF}\times \text{MR}}\;$$

#### 2.4.4 Photosynthetic capability determination

Meanwhile, the chlorophyll content of the leaves aforementioned brought back to the laboratory (2.4.2) was extracted with ethanol/acetone (v/v, 2:1) and measured by ultraviolet spectrophotometry method (Zhang et al., 2022c). Additionally, ten fresh plants in same parts in each plot were selected on May 18 for monitoring their fully-expanded leaves’ capability at 8:00~10:00 a.m by LI-6400XT photosynthesis measurement system (LI-COR Inc., Lincoln, NE, USA) (Chen et al., 2023).

#### 2.4.5 Agronomic trait determination

Moreover, the total plant length, leaf width and length, and stem diameter ten of aforementioned Taizishen plants in each plot were monitored by ruler on May 18, and its leaf area could calculate as:


(16) Leaf area=0.666×leaf width×leaf length

After the investigation of agronomic traits, these plants were sampled as a whole and brought back to the laboratory for measuring their biomass. Total biomass, underground and above-ground biomass of Taizishen were weighed by electronic balance after drying at low temperature (Zhang et al., 2024).

#### 2.4.6 Root growth and quality determination

On July 18, the tuberous roots of Taizishen were sampled for determining growth and medicinal quality. A vernier scale was used for surveying the length and diameter of 100 tuberous roots in each plot, and the gravimetric and oven-drying methods were applied for determining these tuberous roots’ fresh and dry weight. Moreover, the medicinal quality of tuberous roots including extractum, ash, polysaccharide, and total saponins were analyzed as according to the detailed detection methods of Chinese Pharmacopoeia 2020 (2019).

### 2.5 Statistical analyses

The mean ± standard deviation (SD) of three replicates was indicated as data. The significance analysis of the difference between the mean values of the three replicates of every treatments was performed by a one-way analysis of variance (ANOVA) with Duncan’s test, and the normality of the data was performed by the quantile–quantile (Q–Q) plot test. SPSS 18.0 software (SPSS Inc., Chicago, IL, USA) was used for analyzing variance and normality of data. Pearson’s correlation analysis was used for creating a correlation matrix between the disease control efficacy and other parameters. Origin 10.0 was used for drawing figures.

## 3 Results

### 3.1 Influences of tebuconazole·azoxystrobin and tetramycin against leaf spot disease in Taizishen

The controlling effects of tebuconazole·azoxystrobin and tetramycin on leaf spot disease are displayed in **Table 2**. TA 2000+TE 1000, TA 1500, TA 2000, TE 500, and TE 1000 significant (*P* < 0.05) decreased disease index compared with the control. Regarding leaf spot disease, tebuconazole·azoxystrobin displayed an excellent controlling ability; the controlling effects of TA 1500 and TA 2000 could reach 78.45%~76.17% and 67.74%~66.15% at 15 days and 30 days after the final fungicide application, respectively. Meanwhile, tetramycin also showed promising potential in controlling leaf spot disease; the controlling effects of TE 500 and TE 1000 were 75.41%~80.55% and 74.81%~80.26% at 15 days and 30 days after the final fungicide application, respectively. Synergistically, TA 2000+TE 1000 displayed the best control of leaf spot disease, with an effect of 90.03%~90.46% at 15~30 days after the final fungicide application, which is significant (*P* < 0.05) greater than those of TA 1500, TA 2000, TE 500, and TE 1000. These results indicate that low-dosage and persistent tetramycin, combined with quick-acting, persistent, and low-dosage tebuconazole·azoxystrobin, could more efficiently control leaf spot disease in Taizishen compared with high-dosage tebuconazole·azoxystrobin and tetramycin. Moreover, the effective ingredient dosages of fungicide per 667 m^2^ for TA 2000+TE 1000, TA 1500, TA 2000, TE 500, and TE 1000 were 68.04 g, 90 g, 67.5 g, 1.08 g, and 0.54 g, respectively, indicating that the co-application of tebuconazole·azoxystrobin and tetramycin effectively reduced application dosage.

**Table 2:** The control effects of tebuconazole·azoxystrobin and tetramycin on leaf spot disease of Taizishen (*P. heterophylla*) at 15 and 30 days post-treatment under greenhouse conditions.

Treatments	15 days after applying fungicide		30 days after applying fungicide	
	Disease Index	Control effect (%)	Disease Index	Control effect (%)
Control	5.46 ± 0.14 ^a^		6.23 ± 0.20 ^a^	
TE 500	1.06 ± 0.10 ^c^	80.55 ± 2.24 ^c^	1.23 ± 0.07 ^c^	80.26 ± 0.97 ^c^
TE 1000	1.34 ± 0.11 ^b^	75.41 ± 2.69 ^d^	1.57 ± 0.08 ^b^	74.81 ± 0.62 ^d^
TA 1500	0.73 ± 0.08 ^d^	86.60 ± 1.61 ^b^	0.88 ± 0.10 ^d^	85.87 ± 1.58 ^b^
TA 2000	0.98 ± 0.10 ^c^	82.02 ± 2.10 ^c^	1.16 ± 0.04 ^c^	81.36 ± 1.21 ^c^
TA 2000+TE 1000	0.52 ± 0.08 ^e^	90.46 ± 1.55 ^a^	0.62 ± 0.04 ^e^	90.03 ± 1.01 ^a^
*F* values	992.065	22.321	1258.831	79.671
*P* values	<0.001	<0.001	<0.001	<0.001

Different lower-case letters represent statistically significant differences among treatments based on Duncan’s test (*P* < 0.05). Control: water without fungicide. TE 500 and TE 1000: 0.3% tetramycin AS 500-time diluent or 1000-time diluent, respectively. TA 1500 and TA 2000: 75% tebuconazole·azoxystrobin WDG 1500-time diluent or 2000-time diluent, respectively. TA 2000+TE 1000: 75% tebuconazole·azoxystrobin WDG 2000-time + 0.3% tetramycin AS 1000-time diluent.

### 3.2 Influences of tebuconazole·azoxystrobin and tetramycin against viral disease in Taizishen

The controlling effects of tebuconazole·azoxystrobin and tetramycin on viral disease are shown in **Table 3**. Similarly, TA 2000+TE 1000, TA 1500, TA 2000, TE 500, and TE 1000 obviously (*P* < 0.05) declined the incidence rate of viral disease compared with the control. The incidence rate of viral disease with TA 2000+TE 1000 treatment at 15 days after the final fungicide application was significant (*P* < 0.05) lower than that of TA 2000, TE 500, and TE 1000; that treated with TA 2000+TE 1000 at 30 days after the final fungicide application was significant (*P* < 0.05) lower than that of TA 1500, TA 2000, TE 500, and TE 1000. The controlling effect of viral disease under TA 2000+TE 1000 treatment at 15~30 days after the final fungicide application was 71.67%~73.08%, which was significant (*P* < 0.05) higher than that of TA 2000 and TE 1000, but only slightly higher than that of TA 1500 and TE 500. These findings suggest that the co-application of low-dosage tetramycin and tebuconazole·azoxystrobin also could more efficiently control viral disease in Taizishen compared with high-dosage tebuconazole·azoxystrobin and tetramycin, as well as reliably decreasing tebuconazole·azoxystrobin use dosage.

**Table 3:** The control effects of tebuconazole·azoxystrobin and tetramycin on viral disease of Taizishen (*P. heterophylla*) at 15 and 30 days post-treatment under greenhouse conditions.

Treatments	15 days after applying fungicide		30 days after applying fungicide	
	Incidence rate (%)	Control effect (%)	Incidence rate (%)	Control effect (%)
Control	12.67 ± 0.58 ^a^		15.33 ± 0.58 ^a^	
TE 500	4.00 ± 0.00 ^b^	67.52 ± 1.48 ^ab^	5.00 ± 0.00 ^b^	67.36 ± 1.20 ^ab^
TE 1000	4.33 ± 0.58 ^b^	64.74 ± 5.70 ^b^	5.33 ± 0.58 ^b^	65.14 ± 4.57 ^b^
TA 1500	3.67 ± 0.58 ^c^	70.30 ± 4.27 ^ab^	5.00 ± 1.00 ^b^	67.36 ± 1.20 ^ab^
TA 2000	4.33 ± 0.58 ^b^	64.74 ± 5.70 ^b^	5.67 ± 0.58 ^b^	63.06 ± 3.37 ^b^
TA 2000+TE 1000	3.33 ± 0.58 ^c^	73.08 ± 3.33 ^a^	4.33 ± 0.58 ^c^	71.67 ± 4.41 ^a^
*F* values	99.145	3.771	220.029	2.955
*P* values	<0.001	0.040	<0.001	0.045

Different lower-case letters represent statistically significant differences among treatments based on Duncan’s test (*P* < 0.05). Control: water without fungicide. TE 500 and TE 1000: 0.3% tetramycin AS 500-time diluent or 1000-time diluent, respectively. TA 1500 and TA 2000: 75% tebuconazole·azoxystrobin WDG 1500-time diluent or 2000-time diluent, respectively. TA 2000+TE 1000: 75% tebuconazole·azoxystrobin WDG 2000-time + 0.3% tetramycin AS 1000-time diluent.

### 3.3 Influences of tebuconazole·azoxystrobin and tetramycin on MDA, total soluble flavonoids, total soluble phenols, and protective enzyme activity in Taizishen

**Figure 1:**
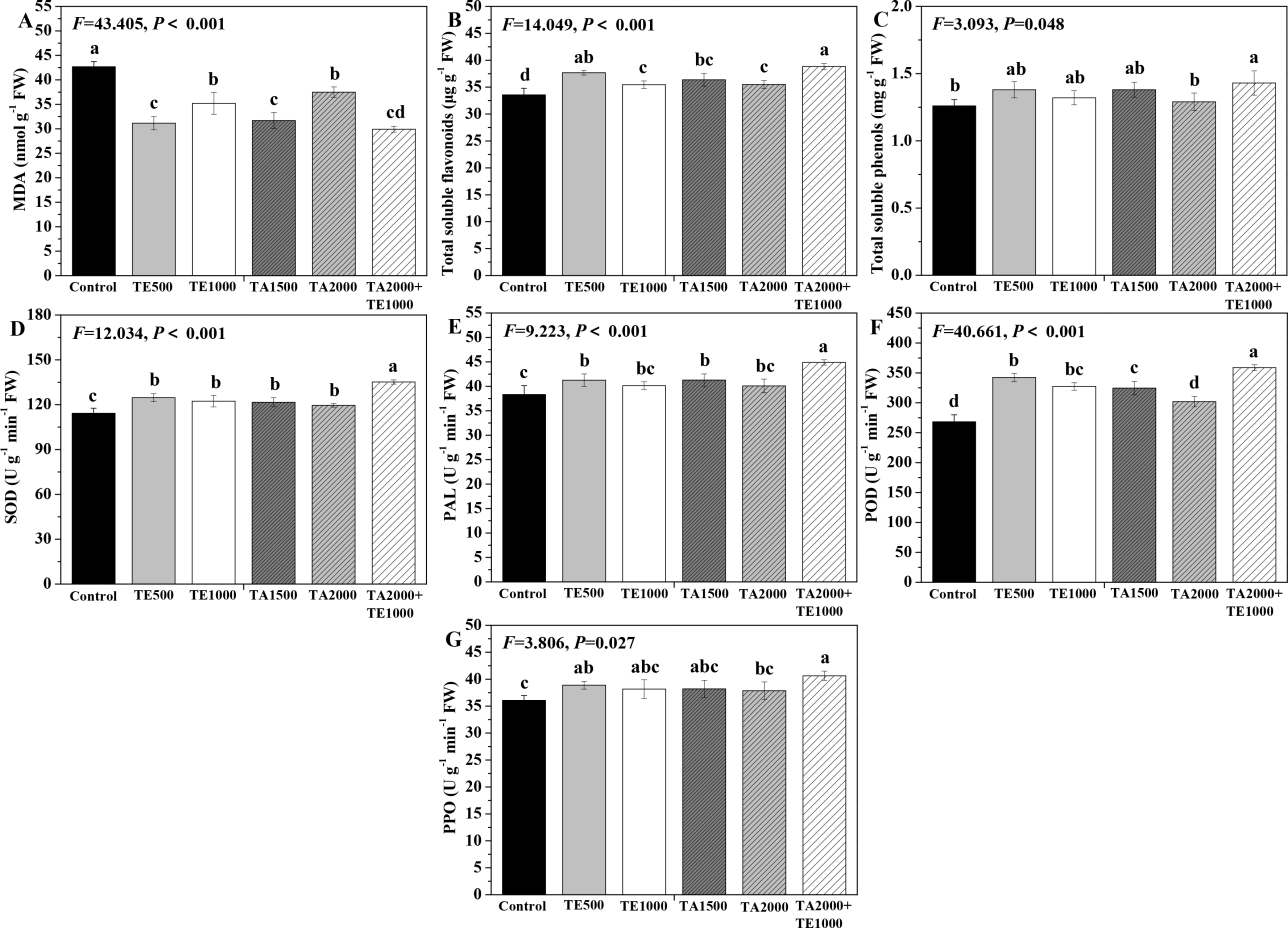
The effect of tebuconazole·azoxystrobin and tetramycin on the malondialdehyde (MDA) and some non-enzymatic and enzymatic antioxidants of Taizishen (*P. heterophylla*) leaves infected with leaf spot and viral diseases at 30 days post-treatment under greenhouse conditions. **(A)** MDA content (nmol g^-1^ FW), **(B)** Total soluble flavonoids content (μg g^-1^ FW), **(C)** total soluble phenolics content (mg g^-1^ FW), **(D)** Superoxide dismutase (SOD) activity (U g^-1^ min^-1^ FW), **(E)** phenylalanine ammonia-lyase (PAL) activity (U g^-1^ min^-1^ FW), **(F)** Peroxidase (POD) activity (UNITS), **(G)** polyphenol oxidase (PPO) activity (U g^-1^ min^-1^ FW). Bars denote the mean of three biological replicates (n=3), whereas error bars show the standard deviation. Different lower-case letters represent statistically significant differences among treatments based on Duncan’s test (*P* < 0.05). Control: water without fungicide. TE 500 and TE 1000: 0.3% tetramycin AS 500-time diluent or 1000-time diluent, respectively. TA 1500 and TA 2000: 75% tebuconazole·azoxystrobin WDG 1000-time diluent or 2000-time diluent, respectively. TA 2000+TE 1000: 75% tebuconazole·azoxystrobin WDG 2000-time + 0.3% tetramycin AS 1000-time diluent.

The effects of tebuconazole·azoxystrobin and tetramycin on the MDA, total soluble flavonoids and total soluble phenols conments of Taizishen are depicted in **Figure 1**. TA 2000+TE 1000, TA 1500, and TE 500 significant (*P* < 0.05) increased the soluble protein content of Taizishen leaves at 30 days post-treatment compared with the control, while TA 2000 and TE 1000 had a slight effect on the soluble protein in leaves. Meanwhile, TA 2000+TE 1000, TA 1500, TA 2000, TE 500, and TE 1000 significant (*P* < 0.05) decreased the leaf MDA content at 30 days post-treatment and improved the leaf total soluble flavonoids content compared with control. Moreover, TA 2000+TE 1000 significant (*P* < 0.05) increased the leaf total soluble phenols content at 30 days post-treatment compared with both the control and TA 2000. Furthermore, the MDA content of leaves treated with TA 2000+TE 1000 at 30 days post-treatment was significant (*P* < 0.05) lower than that of TA 2000 and TE 1000 and slightly lower than that of TA 1500 and TE 500; their total soluble flavonoids content was significant (*P* < 0.05) higher than that of TA 1500, TA 2000, and TE 1000 and slightly higher than that of TE 500; and their total soluble phenols content was significant (*P* < 0.05) greater than that of TA 2000 and slightly higher than that of TA 1500, TE 500, and TE 1000. These results highlight how the combined application of low-dosage tetramycin and tebuconazole·azoxystrobin could more effectively promote the total soluble flavonoids and total soluble phenols of Taizishen than their high-dosage alone application.

The effects of tebuconazole·azoxystrobin and tetramycin on the SOD, PAL, POD, and PPO activities of Taizishen are displayed in **Figure 1**. Compared with the control, TA 2000+TE 1000, TA 1500, TA 2000, TE 500, and TE 1000 could significant (*P* < 0.05) enhance the SOD and POD activities of Taizishen leaves at 30 days post-treatment, TA 2000+TE 1000, TA 1500, and TE 500 could significant (*P* < 0.05) improve their PAL activity, and A 2000+TE 1000 and TE 500 could significant (*P* < 0.05) promote their PPO activity. Furthermore, under TA 2000+TE 1000 treatment at 30 days post-treatment, the leaf SOD, PAL, and POD activities were significant (*P* < 0.05) higher than those of TA 1500, TA 2000, TE 500, and TE 1000. Under TA 2000+TE 1000 treatment at 30 days post-treatment, the PPO activity was significant (*P* < 0.05) higher than that of TA 2000 and slightly higher than that of TA 1500, TE 500, and TE 1000. Additionally, under TA 1500 or TE 500 treatment at 30 days post-treatment, leaf SOD, PAL, POD, and PPO activities were, respectively, slightly higher than those of TA 2000 or TE 1000; under TA 1500, TA 2000, TE 500, and TE 1000 treatments, these functions showed no significant (*P* < 0.05) differences. These results further suggest that tetramycin combined with tebuconazole·azoxystrobin could also obviously enhance the promoting roles of tetramycin or tebuconazole·azoxystrobin alone in the protective enzyme activities of Taizishen at 30 days post-treatment.

### 3.4 Influences of tebuconazole·azoxystrobin and tetramycin on the electrical signals, intracellular water metabolism, nutrient transport, and plant metabolic activity of Taizishen

The influences of tebuconazole·azoxystrobin and tetramycin on the IC, IR, IZ, IXL, and IXc of Taizishen are displayed in **Table 4**. Compared with the control, TA 2000+TE 1000, TA 1500, TA 2000, TE 500, and TE 1000 significant (*P* < 0.05) increased the IC of Taizishen leaves at 30 days post-treatment, effectively decreased their IR, and significantly (*P* < 0.05) decreased their IXL. In addition, TA 2000+TE 1000, TA 1500, and TA 2000 significant (*P* < 0.05) decreased their IZ and IXc at 30 days post-treatment, both of which TE 500 and TE 1000 slightly decreased. Furthermore, under TA 2000+TE 1000 treatment at 30 days post-treatment, leaf IC was significant (*P* < 0.05) greater than that of TA 1500, TA 2000, TE 500, and TE 1000; under TA 2000+TE 1000 treatment, the IR was lower than that of TA 2000, TE 500, and TE 1000. Additionally, under TA 2000+TE 1000 treatment at 30 days post-treatment, IZ and IXc were significant (*P* < 0.05) lower than those of TE 500 and TE 1000, with no significant (*P* < 0.05) differences between TA 1500 and TA 2000. Moreover, under TA 1500 or TE 500 treatment at 30 days post-treatment, leaf IC was, respectively, higher than that of TA 2000 or TE 1000, and IR, IZ, IXL, and IXc were, respectively, lower than those of TA 2000 or TE 1000. These results show that the combined application of low-dosage tetramycin and tebuconazole·azoxystrobin could more effectively promote the IC of Taizishen, decreasing its IR, IZ, IXL, and IXc compared to their high-dosage, singular use and more effectively promoting its growth.

**Table 4:** The influences of tebuconazole·azoxystrobin and tetramycin on the IC, IR, IZ, IXL, and IXc of Taizishen (*P. heterophylla*) leaves infected with leaf spot and viral diseases at 30 days post-treatment under greenhouse conditions.

Treatments	IC (pF)	IR (MΩ)	IZ (MΩ)	IXL (MΩ)	IXc (MΩ)
Control	161.39 ± 19.06 ^d^	0.65 ± 0.37 ^a^	0.27 ± 0.07 ^a^	0.33 ± 0.04 ^a^	0.85 ± 0.37 ^a^
TE 500	216.51 ± 23.57 ^d^	0.63 ± 0.44 ^a^	0.22 ± 0.03 ^a^	0.25 ± 0.03 ^b^	0.76 ± 0.33 ^a^
TE 1000	191.67 ± 23.81 ^d^	0.85 ± 0.39 ^a^	0.25 ± 0.07 ^a^	0.28 ± 0.04 ^b^	1.02 ± 0.39 ^a^
TA 1500	385.89 ± 66.31 ^b^	0.29 ± 0.12 ^b^	0.12 ± 0.03 ^b^	0.14 ± 0.02 ^cd^	0.36 ± 0.13 ^b^
TA 2000	313.73 ± 14.45 ^c^	0.37 ± 0.22 ^ab^	0.14 ± 0.03 ^b^	0.17 ± 0.01 ^c^	0.46 ± 0.12 ^b^
TA 2000+TE 1000	482.07 ± 16.41 ^a^	0.33 ± 0.18 ^ab^	0.10 ± 0.01 ^b^	0.11 ± 0.00 ^d^	0.36 ± 0.13 ^b^
*F* values	44.280	0.958	8.230	37.035	1.512
*P* values	<0.001	0.044	0.001	<0.001	0.046

Different lower-case letters represent statistically significant differences among treatments based on Duncan’s test (*P* < 0.05). Control: water without fungicide. TE 500 and TE 1000: 0.3% tetramycin AS 500-time diluent or 1000-time diluent, respectively. TA 1500 and TA 2000: 75% tebuconazole·azoxystrobin WDG 1500-time diluent or 2000-time diluent, respectively. TA 2000+TE 1000: 75% tebuconazole·azoxystrobin WDG 2000-time + 0.3% tetramycin AS 1000-time diluent.

**Figure 2:**
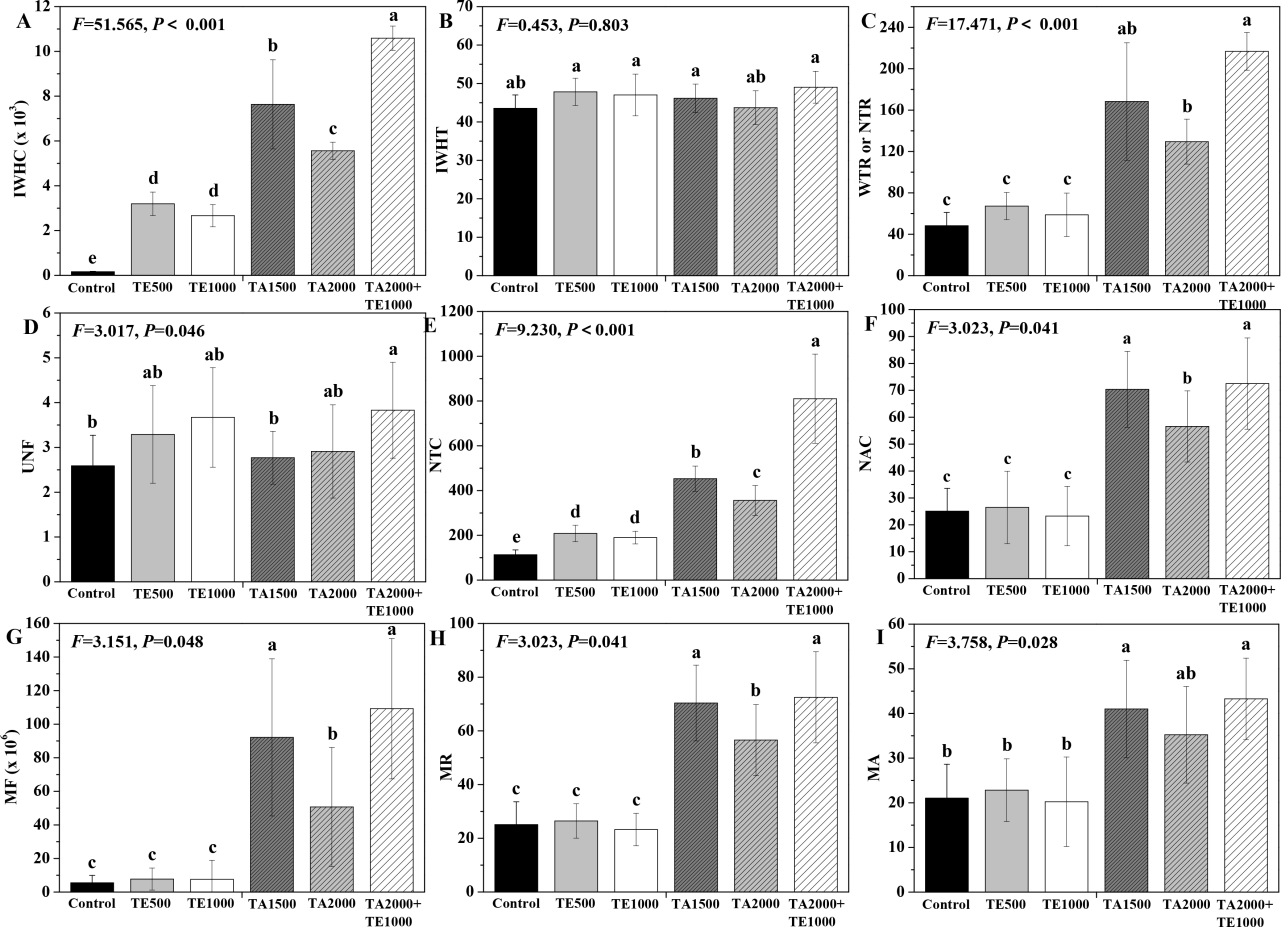
The effects of tebuconazole·azoxystrobin and tetramycin on the intracellular water metabolism, nutrient transport, and metabolic activity of Taizishen (*P. heterophylla*) leaves infected with leaf spot and viral diseases at 30 days post-treatment under greenhouse conditions. **(A)** Intracellular water-holding capacity (IWHC), **(B)** Intracellular water-holding time (IWHT), **(C)** Water or nutrient transfer rate (WTR or NTR), **(D)** Nutrient flux per unit area (UNF), **(E)** Nutrient transport capacity (NTC), **(F)** Nutrient active transport capacity (NAC), **(G)** Metabolic flow (MF), **(H)** Metabolic rate (MR), and **(I)** Metabolic activity (MA). Bars denote the mean of three biological replicates (n=3), whereas error bars show the standard deviation. Different lower-case letters represent statistically significant differences among treatments based on Duncan’s test (*P* < 0.05). Control: water without fungicide. TE 500 and TE 1000: 0.3% tetramycin AS 500-time diluent or 1000-time diluent, respectively. TA 1500 and TA 2000: 75% tebuconazole·azoxystrobin WDG 1000-time diluent or 2000-time diluent, respectively. TA 2000+TE 1000: 75% tebuconazole·azoxystrobin WDG 2000-time + 0.3% tetramycin AS 1000-time diluent.

The influences of tebuconazole·azoxystrobin and tetramycin on the intracellular water metabolism, nutrient transport, and metabolic activity of Taizishen are displayed in **Figure 2**. In terms of intracellular water metabolism, TA 2000+TE 1000, TA 1500, TA 2000, TE 500, and TE 1000 significant (*P* < 0.05) enhanced IWHC at 30 days post-treatment compared with the control and TA 2000+TE 1000, TA 1500, and TA 2000 significant (*P* < 0.05) increased WTR or NTR. Although these treatments could also improve IWHT, there were no differences between them. Moreover, the IWHC of Taizishen treated with TA 2000+TE 1000 at 30 days post-treatment was significant (*P* < 0.05) higher than that of TA 1500, TA 2000, TE 500, and TE 1000; the WTR or NTR treated with TA 2000+TE 1000 was also significant (*P* < 0.05) higher than that of TA 2000, TE 500, and TE 1000. For the nutrient transport status of Taizishen at 30 days post-treatment, TA 2000+TE 1000 significant (*P* < 0.05) enhanced UNF compared with the control, and TA 2000+TE 1000, TA 1500, TA 2000, TE 500, and TE 1000 significant (*P* < 0.05) improved NTC; in addition, TA 2000+TE 1000, TA 1500, and TA 2000 significant (*P* < 0.05) promoted NAC. Meanwhile, the UNF of Taizishen treated with TA 2000+TE 1000 at 30 days post-treatment was significant (*P* < 0.05) higher than that of TA 1500 and obviously higher than that of TA 2000, TE 500, and TE 1000; the NTC treated with TA 2000+TE 1000 at 30 days post-treatment was significant (*P* < 0.05) higher than that of TA 1500, TA 2000, TE 500, and TE 1000, and the NAC treated with TA 2000+TE 1000 at 30 days post-treatment was significant (*P* < 0.05) greater than that of TA 2000, TE 500, and TE 1000 and slight higher than that of TA 1500. Regarding the metabolic activity of Taizishen at 30 days post-treatment, TA 2000+TE 1000, TA 1500, and TA 2000 significant (*P* < 0.05) improved the MF, MR, and MA compared with the control, and TE 500 and TE 1000 also slightly promoted these properties. Nevertheless, the MF and MR of Taizishen treated with TA 2000+TE 1000 at 30 days post-treatment were significant (*P* < 0.05) higher than those of TA 2000, TE 500, and TE 1000 and slightly higher than those of TA 1500; the MA treated with TA 2000+TE 1000 was significant (*P* < 0.05) higher than that of TE 500 and TE 1000 and slightly higher than that of TA 1500 and TA 2000. These findings demonstrate that low-dosage tetramycin and tebuconazole·azoxystrobin effectively enhance the intracellular water metabolism, nutrient transport, and plant metabolic activity of Taizishen, improving its healthy growth. Furthermore, their low-dosage co-application was even more effective.

### 3.5 Influences of tebuconazole·azoxystrobin and tetramycin on the photosynthetic capability and agronomic traits of Taizishen

**Figure 3:**
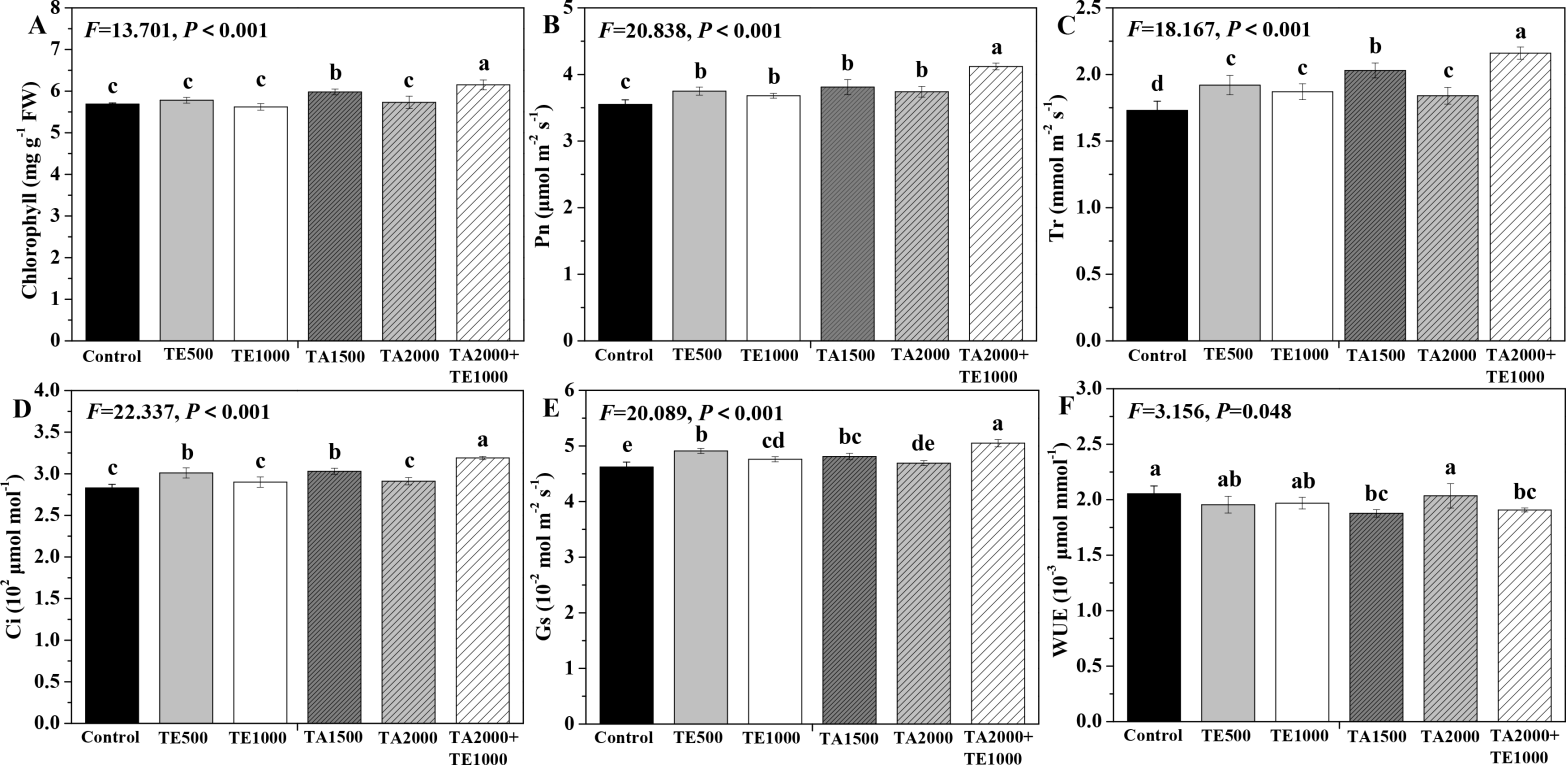
The effects of tebuconazole·azoxystrobin and tetramycin on the photosynthetic capability of Taizishen (*P. heterophylla*) leaves infected with leaf spot and viral diseases at 30 days post-treatment under greenhouse conditions. **(A)** chlorophyll content (mg g^-1^ FW), **(B)** photosynthetic rate (Pn) (μmol m^-2^ s^-1^), **(C)** transpiration rate (Tr) (mmol m^-2^ s^-1^), **(D)** intercellular carbon dioxide concentration (Ci) (μmol mol^-1^), **(E)** stomatal conductance (Gs) (10^-2^ mol m^-2^ s^-1^), and **(F)** water use efficiency (WUE) (10^-3^ μmol mmol^-1^). Different lower-case letters represent statistically significant differences among treatments based on Duncan’s test (*P* < 0.05). Control: water without fungicide. TE 500 and TE 1000: 0.3% tetramycin AS 500-time diluent or 1000-time diluent, respectively. TA 1500 and TA 2000: 75% tebuconazole·azoxystrobin WDG 1000-time diluent or 2000-time diluent, respectively. TA 2000+TE 1000: 75% tebuconazole·azoxystrobin WDG 2000-time + 0.3% tetramycin AS 1000-time diluent.

The influences of tebuconazole·azoxystrobin and tetramycin on the photosynthetic capability of Taizishen are depicted in **Figure 3**. Compared with the control, TA 2000+TE 1000 and TA 1500 significant (*P* < 0.05) enhanced the chlorophyll content of Taizishen at 30 days post-treatment and decreased its WUE. TA 2000+TE 1000, TA 1500, TA 2000, TE 500, and TE 1000 significant (*P* < 0.05) improved Pn and Tr, and TA 2000+TE 1000, TA 1500, and TE 500 significant (*P* < 0.05) increased Ci; furthermore, TA 2000+TE 1000, TA 1500, TE 500, and TE 1000 significant (*P* < 0.05) promoted Gs. Additionally, the leaf chlorophyll, Pn, Tr, Ci, and Gs of Taizishen treated by TA 2000+TE 1000 at 30 days post-treatment were significant (*P* < 0.05) higher: 1.01, 1.05, 1.04, and 1.07 times; 1.06, 1.08, 1.07, and 1.10 times; 1.06, 1.17, 1.12, and 1.15 times; 1.05, 1.09, 1.06, and 1.10 times; and 1.05, 1.02, 1.02, and 1.06 times compared with TA 1500, TA 2000, TE 500, and TE 1000, respectively. Meanwhile, the leaf chlorophyll, Pn, Tr, Ci, and Gs of Taizishen treated with TA 1500 or TE 500 at 30 days post-treatment were higher than those of TA 2000 or TE 1000, respectively. These results show that the combined application of low-dosage tetramycin and tebuconazole·azoxystrobin could more effectively improve the photosynthetic capacity of Taizishen than their high-dosage application alone. In addition, this combination could more reliably ameliorate growth.

**Figure 4:**
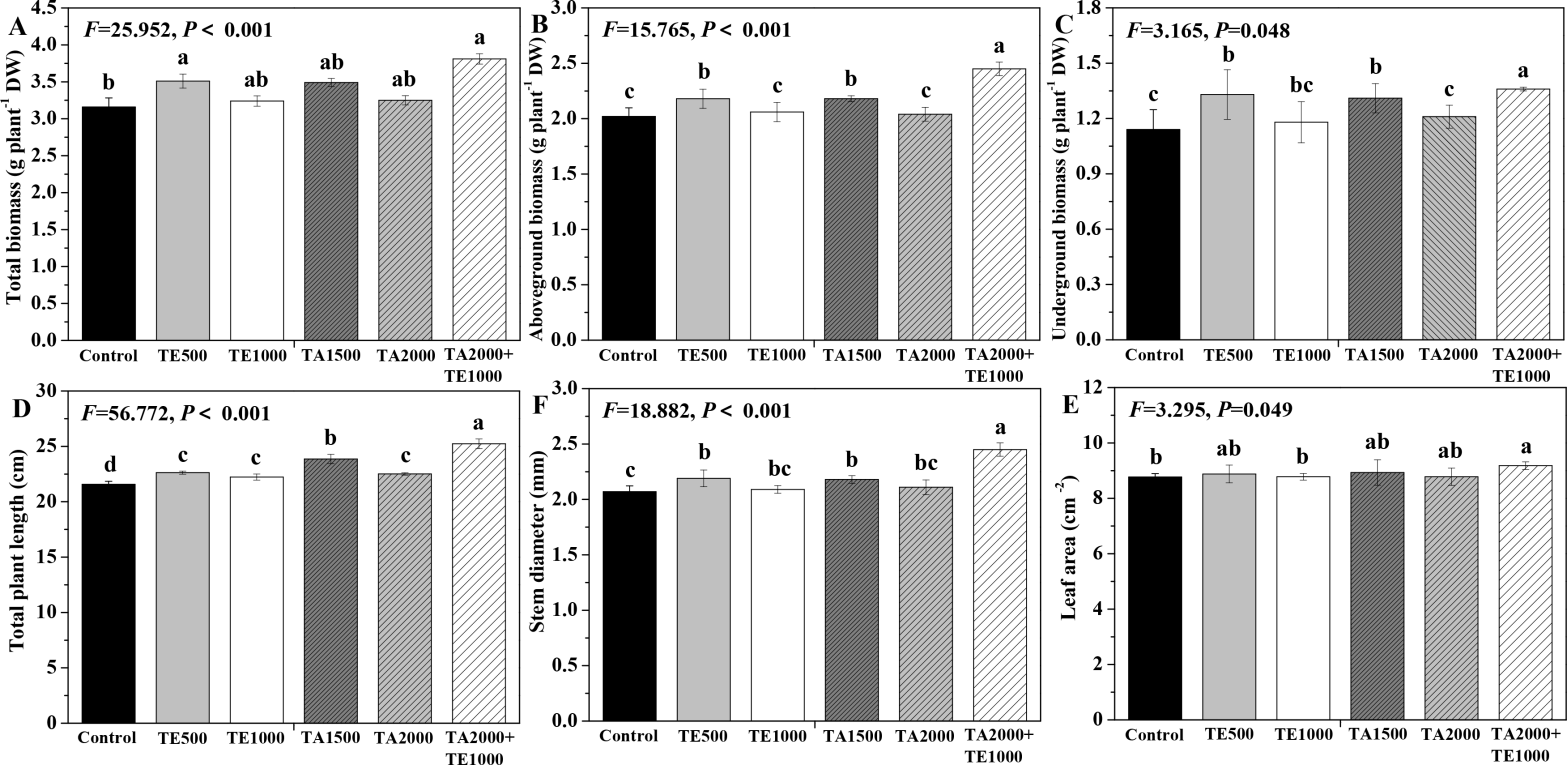
The effects of tebuconazole·azoxystrobin and tetramycin on the agronomic traits of Taizishen (*P. heterophylla*) plants infected with leaf spot and viral diseases at 30 days post-treatment under greenhouse conditions. **(A)** total biomass (g plant^-1^ DW), **(B)** aboveground biomass (g plant^-1^ DW), **(C)** underground biomass (g plant^-1^ DW), **(D)** plant length (cm), **(E)** leaf area (cm^2^), and **(F)** stem diameter (cm). Different lower-case letters represent statistically significant differences among treatments based on Duncan’s test (*P* < 0.05). Control: water without fungicide. TE 500 and TE 1000: 0.3% tetramycin AS 500-time diluent or 1000-time diluent, respectively. TA 1500 and TA 2000: 75% tebuconazole·azoxystrobin WDG 1000-time diluent or 2000-time diluent, respectively. TA 2000+TE 1000: 75% tebuconazole·azoxystrobin WDG 2000-time + 0.3% tetramycin AS 1000-time diluent.

The influences of tebuconazole·azoxystrobin and tetramycin on the biomass and agronomic traits of Taizishen are depicted in **Figure 4**. Compared with the control, TA 2000+TE 1000 and TE 500 significant (*P* < 0.05) enhanced the total biomass of Taizishen at 30 days post-treatment; TA 2000+TE 1000, TA 1500, and TE 500 significant (*P* < 0.05) increased its above- and underground biomass and stem diameter at 30 days post-treatment; TA 2000+TE 1000, TA 1500, TA 2000, TE 500, and TE 1000 significant (*P* < 0.05) increased its plant length at 30 days post-treatment; and TA 2000+TE 1000 also significant (*P* < 0.05) increased its leaf area at 30 days post-treatment. Meanwhile, the above- and underground biomass, plant length, and stem diameter of Taizishen treated with TA 2000+TE 1000 at 30 days post-treatment were significant (*P* < 0.05) higher than those of TA 1500, TA 2000, TE 500, and TE 1000. The findings demonstrate that low-dosage tetramycin, when used together with low-dosage tebuconazole·azoxystrobin, could more effectively promote the biomass, agronomic traits, and growth of Taizishen than their high-dosage application alone.

### 3.6 Influences of tebuconazole·azoxystrobin and tetramycin on yield and quality of Taizishen

**Figure 5:**
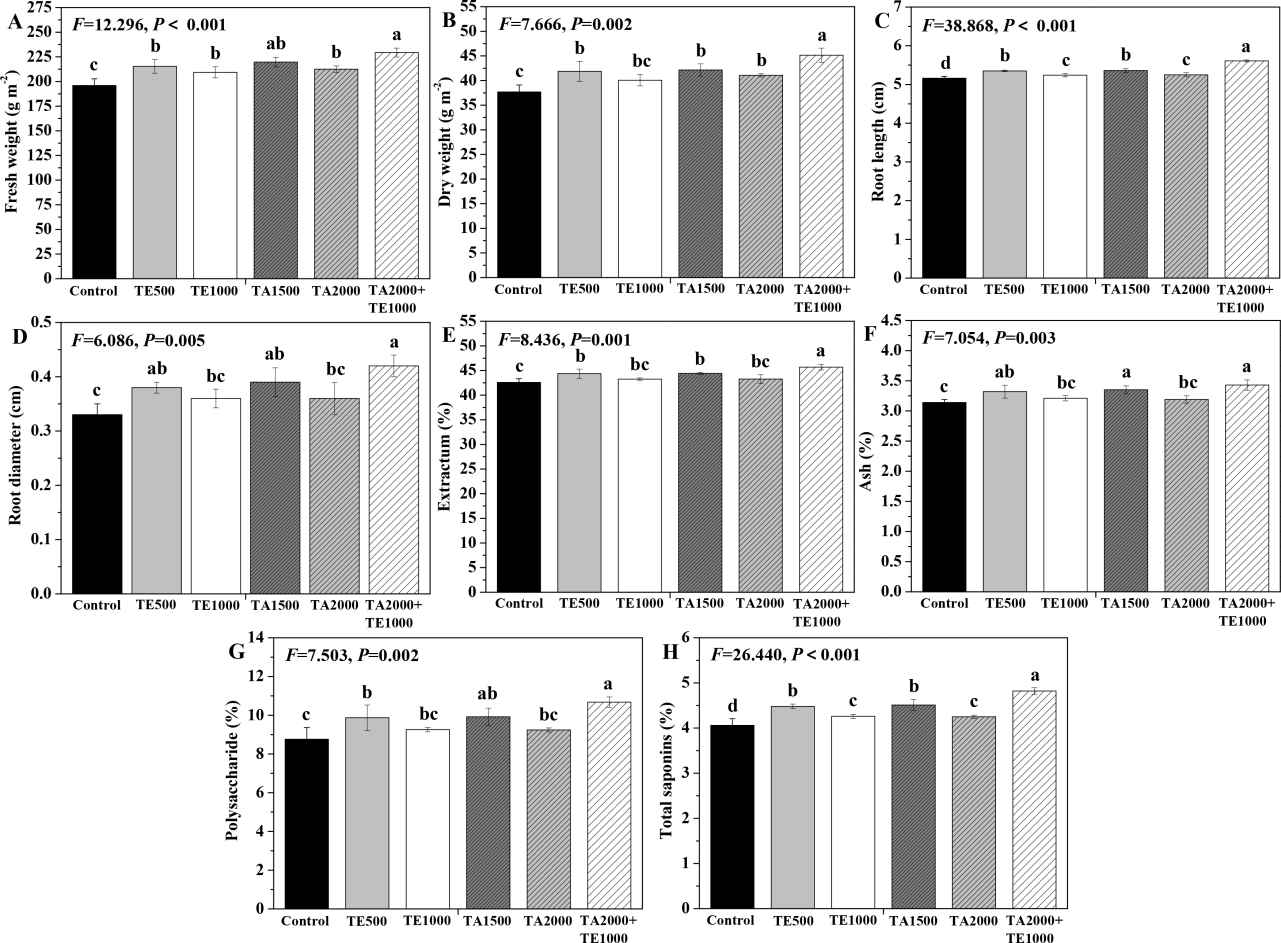
The effects of tebuconazole·azoxystrobin and tetramycin on the yield and quality of Taizishen (*P. heterophylla*) plants infected with leaf spot and viral diseases at 90 days post-treatment under greenhouse conditions. **(A)** fresh weight (g m^-2^), **(B)** dry weight (g m^-2^), **(C)** root length (cm), **(D)** root diameter (cm), **(E)** extractum (%), **(F)** ash (%), **(G)** polysaccharide (%), and **(H)** total saponins (%). Different lower-case letters represent statistically significant differences among treatments based on Duncan’s test (*P* < 0.05). Control: water without fungicide. TE 500 and TE 1000: 0.3% tetramycin AS 500-time diluent or 1000-time diluent, respectively. TA 1500 and TA 2000: 75% tebuconazole·azoxystrobin WDG 1000-time diluent or 2000-time diluent, respectively. TA 2000+TE 1000: 75% tebuconazole·azoxystrobin WDG 2000-time + 0.3% tetramycin AS 1000-time diluent.

The influences of tebuconazole·azoxystrobin and tetramycin on the fresh and dry weights, as well as the root length and diameter, are depicted in **Figure 5**. Compared with control, TA 2000+TE 1000, TA 1500, TA 2000, and TE 500 significant (*P* < 0.05) increased the fresh and dry weights and root length and diameter of Taizishen roots at 90 days post-treatment, while TE 1000 also significant (*P* < 0.05) enhanced their fresh weight and root length. Moreover, the fresh weight of Taizishen treated with TA 2000+TE 1000 at 90 days post-treatment was significant (*P* < 0.05) higher than that of TA 2000, TE 500, and TE 1000, and its dry weight and root length under TA 2000+TE 1000 treatment at 90 days post-treatment were significant (*P* < 0.05) higher than those of TA 1500, TA 2000, TE 500, and TE 1000. Furthermore, under TA 2000+TE 1000 treatment at 90 days post-treatment, the root diameter was significant (*P* < 0.05) higher than that of TA 2000 and TE 1000 alone. These results reveal that the ameliorating effects of the combination of low-dosage tebuconazole·azoxystrobin and tetramycin on Taizishen root growth and weight were better than those of singular, high doses.

The influences of tebuconazole·azoxystrobin and tetramycin on the medicinal quality of Taizishen roots are depicted in **Figure 5**. Compared with the control, TA 2000+TE 1000, TA 1500, and TE 500 significant (*P* < 0.05) improved the extractum, ash, polysaccharide, and total saponins of Taizishen roots at 90 days post-treatment, and TA 2000 and TE 1000 also significant (*P* < 0.05) increased their total saponins. Simultaneously, the extractum and total saponins of Taizishen roots under TA 2000+TE 1000 treatment at 90 days post-treatment were significant (*P* < 0.05) higher than those of TA 1500, TA 2000, TE 500, and TE 1000; under TA 2000+TE 1000 treatment at 90 days post-treatment, the ash content was significant (*P* < 0.05) higher than that of TA 2000 and TE 1000; and, under TA 2000+TE 1000 treatment at 90 days post-treatment, its polysaccharide content was also significant (*P* < 0.05) greater than that of TA 2000, TE 500, and TE 1000. Moreover, their extractum, ash, polysaccharide, and total saponin contents under TA 2000 or TE 500 treatment at 90 days post-treatment were apparently greater than those of TA 1500 or TE 1000. These results demonstrate that the medicinal quality of Taizishen root was better enhanced by tebuconazole·azoxystrobin + tetramycin than by tebuconazole·azoxystrobin or tetramycin alone.

### 3.7 Correlation analysis

**Figure 6:**
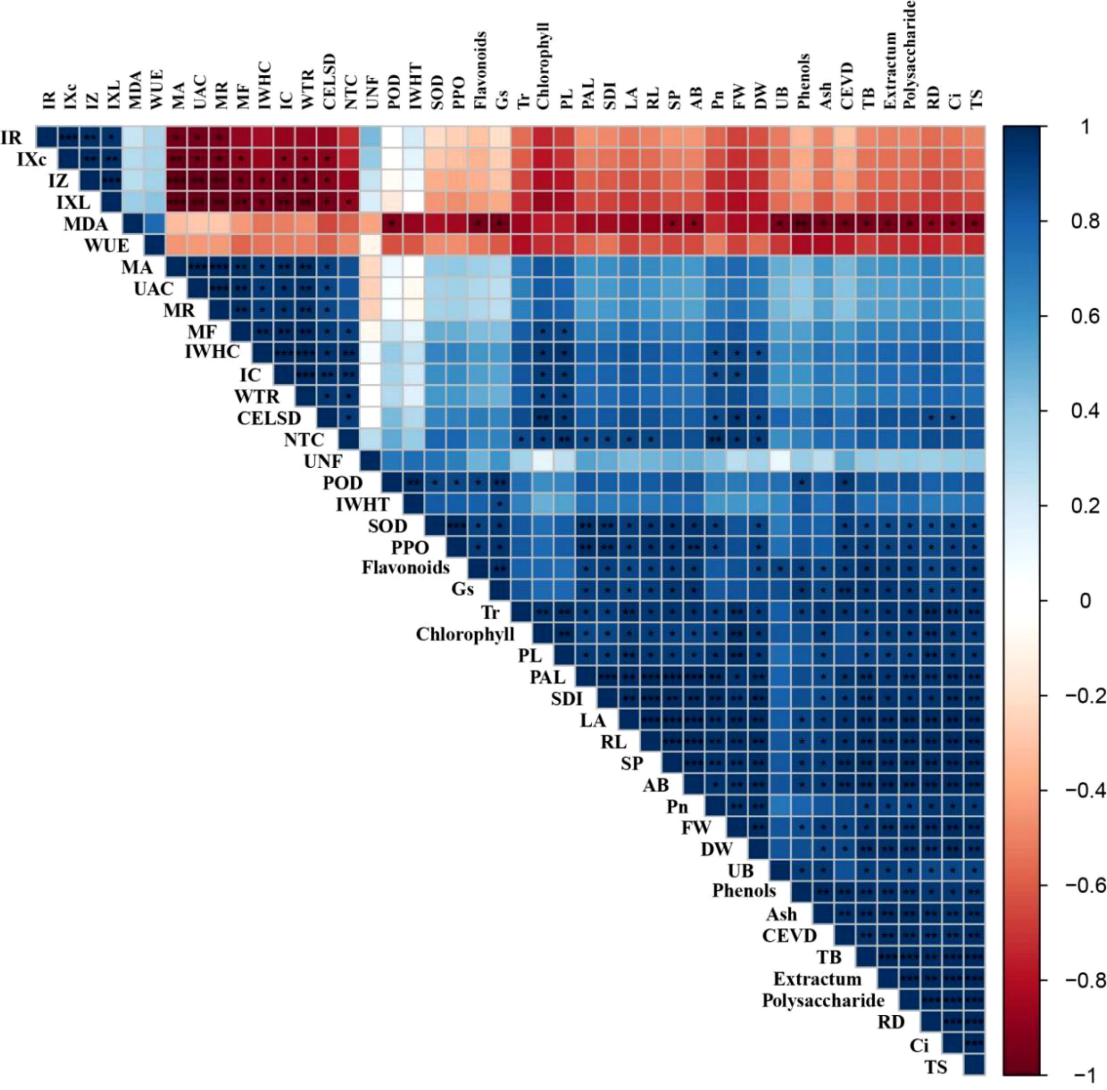
Correlation analysis between control efficacy and resistance, electrophysiology, photosynthesis, growth, and quality parameters. CELSD, control effect of leaf spot disease; CEVD, control effect of viral disease; SP, soluble protein; TB, total biomass; AB, aboveground biomass, UB, underground biomass; PL, plant length; LA, leaf area; SDI, stem diameter; FW, fresh weight; DW, dry weight; RL, root length; RD, root diameter; TS, total saponins. *, **, and *** represent significant correlations at 0.05 (*P* < 0.05), 0.01 (*P* < 0.01), and 0.001 (*P* < 0.001) levels, respectively.

Pearson’s correlation analysis was used for creating a correlation matrix between the disease control efficacy and the resistance, electrophysiology, photosynthesis, growth, and quality parameters of Taizishen. As shown in **Figure 6**, the control effect of leaf spot disease in Taizishen is significant (*P* < 0.05) positive correlated with its MA, UAC, MR, MF, IWHC, WTR, NTC, Pn, plant length, fresh weight, dry weight, underground biomass, and root diameter, and significantly (*P* < 0.01) positive correlated with its IC and chlorophyll, as well as significant (*P* < 0.05) negative correlated with its IXc, IZ, and IXL. Meanwhile, the control effect of viral disease in Taizishen is significant (*P* < 0.05) positive correlated with its POD, SOD, PPO, PAL, total soluble flavonoids, Tr, fresh weight, dry weight and root length, and significantly (*P* < 0.01) positive correlated with its soluble protein, total soluble phenols, Gs, aboveground biomass, and ash, as well as significant (*P* < 0.05) negative correlated with its MDA. These results demonstrate that the control effect of leaf spot disease in Taizishen treated by the combination of low-dosage tebuconazole·azoxystrobin and tetramycin exhibited good correlations with its electrophysiology, photosynthesis, and growth parameters, and the control effect of viral disease in Taizishen treated by the combination of low-dosage tebuconazole·azoxystrobin and tetramycin exhibited good correlations with its disease resistance, photosynthesis, growth, and quality parameters.

## 4 Discussion

The leaf spot pathogens of Taizishen are *Alteraria tenuissima*, *Arcopilus versabilis*, *Phyllosticta commonsii*, etc., and those of viral disease are *Turnip mosaic virus*, *Broad bean wilt virus*, and *Tobacco mosaic virus* (He et al., 2021; Long et al., 2013; Li et al., 2018; Liang et al., 2022; Yang et al., 2023a, 2023). Tebuconazole can inhibit the sterol biosynthesis of numerous pathogenic fungi (Fustinoni et al., 2014; Paul et al., 2008; Liu et al., 2015), and azoxystrobin exerts broad-spectrum systemic activity on several pathogens (Adetutu et al., 2010; Rodrigue et al., 2013; Wang et al., 2016). Additionally, tetramycin can be widely applied for preventing and controlling various plant diseases caused by fungal, bacterial, and viral pathogens (Chen et al., 2017; Gao et al., 2018; Song et al., 2016; Li et al., 2023; Ma et al., 2017, 2018; Zhao et al., 2010). Wang et al. (2021a) reported that tetramycin exhibited superior antifungal activity against *Alternaria tenuissima*, a kiwifruit brown-spot pathogen with an EC_50_ value of 0.16 mg kg^−1^. Moreover, our previous research findings showed that tetramycin, when combined with chitosan or matrine, could reliably control leaf spot or soft rot diseases in kiwifruit, improving its resistance, photosynthesis, and quality (Wang et al., 2021b; Zhang et al., 2022a, 2022b). In this study, TA 2000+TE 1000 displayed the best performance in controlling leaf spot and viral diseases, with effects of 90.03%~90.46% and 71.67%~73.08% at 15~30 days after the final fungicide application, respectively; these values are obviously higher than those of TA 1500, TA 2000, TE 500, and TE 1000. These results demonstrate that low-dosage tetramycin, combined with low-dosage tebuconazole·azoxystrobin, could more efficiently control leaf spot and viral diseases compared with high-dosage tebuconazole·azoxystrobin or tetramycin alone, as well as effectively reduce tebuconazole·azoxystrobin application. The superior antimicrobial activity of tetramycin, and the systemic, protective, and therapeutic characteristics of tebuconazole or azoxystrobin, effectively drove the synergistic effect of low-dosage tetramycin and tebuconazole·azoxystrobin in leaf spot and viral diseases.

Inducing disease resistance is considered a feasible method for controlling plant diseases. MDA, flavonoids, phenols, SOD, PAL, POD, and PPO activities, are actively involved in disease-resistance processes in plants (Vlot et al., 2021; Zhang et al., 2022a). For instance, flavonoids and phenols can enhance host cells’ ligninization by participating in lignin biosynthesis, SOD and POD can alleviate reactive oxygen species damage, and PAL and PPO can participate in lignin or phytoalexins biosynthesis (Vlot et al., 2021). Wang et al. (2021a) found that tetramycin could significant (*P* < 0.01) enhance the phenolic and flavonoid contents, as well as the SOD and PPO activities of kiwifruit, and notably increase fruit disease resistance. In this study, TA 2000+TE 1000 significant (*P* < 0.05) increased the total soluble flavonoids and total soluble phenols contents, SOD, PAL, POD, and PPO activities of Taizishen at 30 days post-treatment, while decreasing its MDA content. Meanwhile, the promoting or inhibitory effects of TA 1500, TA 2000, TE 500, and TE 1000 on these resistance parameters were weaker than those of TA 2000+TE 1000. These results show how the combined application of low-dosage tetramycin and tebuconazole·azoxystrobin could more promote the resistance substance contents and resistance enzyme activities of Taizishen than their high-dosage application alone. In this way, this treatment has promising potential for ameliorating disease resistance in Taizishen.

A plant’s electrophysiological activities run throughout almost all of its life processes; these activities are considered the fastest responses to environmental stresses (Sukhov, 2016; Szechyńska-Hebda et al., 2017; Xing et al., 2022). Abiotic or biotic stress, such as diseases, insect pests, drought, and so on, can directly or indirectly causes dramatic changes in their electrophysiological activities (Sukhov, 2016; Szechyńska-Hebda et al., 2017; Xing et al., 2022; Tian et al., 2024; Zhang et al., 2020, 2021a, 2021b). Previously, it has been shown that C, R, Z, Xc, and XL are the most common electrical signals used to evaluate various plants’ physiological status (Zhang et al., 2015; Xing et al., 2021; Zhang et al., 2020, 2021a, 2021b; Tian et al., 2024). In this work, the co-application of low-dosage tetramycin and tebuconazole·azoxystrobin could more effectively enhance the IC, intracellular water metabolism, nutrient transport, and plant metabolic activity of Taizishen and decline its IR, IZ, IXL, and IXc than their high-dosage, singular application. These results emphasize that the co-application of low-dosage tetramycin and tebuconazole·azoxystrobin could effectively promote healthy growth in Taizishen. This plant’s electrophysiological information can effectively aid in characterizing its physiological activities, thus supporting the research conclusions of the above disease control roles following photosynthesis, biomass, and agronomic traits.

The physiological basis of plant growth and development is photosynthesis, and good growth facilities high biomass and quality. Wang et al. (2021b) reported that tetramycin, when used together with chitosan, could enhance the photosynthesis, growth, and quality of kiwifruit. Similarly, our previous research findings showed that tetramycin combined with matrine could also reliably enhance kiwifruit’s photosynthesis, quality, and amino acid levels (Wang et al., 2021b; Zhang et al., 2022a, 2022b). In this study, compared with high-dosage tetramycin and tebuconazole·azoxystrobin, the combined application of low doses could more promote the chlorophyll, Pn, Tr, Ci, and Gs of Taizishen; more reliably increase its total, above-, and underground biomass, as well as its plant length, leaf area, and stem diameter; and more notably ameliorate its root weight, length, and diameter, as well as more obviously enhance its roots’ extractum, ash, polysaccharide, and total saponin contents. Moreover, the control effect of leaf spot disease in Taizishen treated by the combination of low-dosage tebuconazole·azoxystrobin and tetramycin exhibited significant correlations with its electrophysiology, photosynthesis, and growth parameters, and the control effect of viral disease in Taizishen treated by the combination of low-dosage tebuconazole·azoxystrobin and tetramycin exhibited significant correlations with its MDA, total soluble flavonoids, total soluble phenols, protective enzyme activity, photosynthesis, growth, and quality parameters. These positive results underscore how the co-application of low-dosage tetramycin and tebuconazole·azoxystrobin can effectively control leaf spot and viral diseases in Taizishen, thereby facilitating good growth, high biomass, and an excellent medicinal quality.

In general, reducing the use of chemical pesticides and exploiting alternative technologies are two approaches that will always be favored by the public and the government. Meanwhile, high-efficacy and low-toxicity natural products for use as synergists in managing plant diseases and decreasing chemical pesticide use are being increasingly welcomed (Wang et al., 2022). In this study, the combined application of low-dosage tetramycin and tebuconazole·azoxystrobin could more control leaf spot and viral diseases in Taizishen compared with high-dosage tebuconazole·azoxystrobin or tetramycin alone. This treatment combination could enhance its disease resistance, intracellular water metabolism, nutrient transport, plant metabolic activity, photosynthesis, growth, yield, and quality, and reliably decrease tebuconazole·azoxystrobin application. Furthermore, the safe interval period is very long at over 60 days, and tetramycin is a natural, environmentally friendly, and low-toxicity antibiotic with wide applications in medical, agricultural, and other fields. Thus, the food safety risk with this combined treatment is minuscule to nonexistent. All in all, this work highlights how 75% tebuconazole·azoxystrobin WDG 2000-time + 0.3% tetramycin AS diluted 1000 times is a practicable formula for controlling leaf spot and viral diseases in Taizishen.

## 5 Conclusions

In summary, tetramycin could effectively assist low-dosage tebuconazole·azoxystrobin in protecting against leaf spot and viral diseases in Taizishen. Moreover, the co-application of low-dosage tetramycin and tebuconazole·azoxystrobin could more effectively improve the resistance-related substance contents and resistance enzyme activities of Taizishen than their high-dosage, singular application. Meanwhile, their combined application could more effectively ameliorate the plant’s IC, intracellular water metabolism, nutrient transport, and plant metabolic activity, and decrease its IR, IZ, IXL, and IXc. Additionally, their combined application could also more effectively enhance the plant’s photosynthesis, biomass, agronomic traits, and root growth and quality. This study highlights that the combined application of low-dosage tetramycin and tebuconazole·azoxystrobin can be recommended as a novel and practicable measure for controlling leaf spot and viral diseases in Taizishen.

## Data Availability

The original contributions presented in the study are included in the article/supplementary material. Further inquiries can be directed to the corresponding author/s.
